# Review and outlook of global energy use under the impact of COVID‐19

**DOI:** 10.1002/eng2.12584

**Published:** 2022-11-06

**Authors:** Dongdong Zhang, Cunhao Rong, Tanveer Ahmad, Haonan Xie, Hongyu Zhu, Xiang Li, Thomas Wu

**Affiliations:** ^1^ School of Electrical Engineering Guangxi University Nanning China; ^2^ State Key Laboratory of Internet of Things for Smart City University of Macau Macau China

**Keywords:** CO_2_ emissions, energy consumption, energy outlook, renewable energy

## Abstract

By collecting and sorting the energy demand data of developing and developed countries, this paper makes a comprehensive analysis of their energy demand, including the change of energy demand and the change trend of energy load in various sectors. The survey scope of the article includes the overall change trend of energy supply, natural gas, oil, electricity, coal, renewable energy (such as wind energy, solar energy, geothermal energy, tidal energy, etc.), and the data change of global carbon dioxide emission. Besides, this paper selects a variety of energy sources for comprehensive analysis to analyze the existing change trend in chronological order. The analysis methods include data statistics of primary energy production and consumption, energy intensity analysis of gross domestic product (GDP), production, and demand balance of oil, natural gas, and coal, and study the trade balance between different types of energy in different countries and regions. The regions examined in this review include the organization for economic cooperation and development (OECD); the group of seven (G7); Brazil, Russia, India, China and South Africa (BRICs); the European Union; Europe; North America; the Commonwealth of Independent States (CIS); Asia; Latin America; the Pacific Ocean; the Middle East and Africa. By studying these data, we can make a better summary of the current energy use, so as to conveniently grasp the context of energy development and have a general understanding of the current energy structure. Therefore, individuals and policymakers in the fields of energy trade can think more deeply about the future situation and draw conclusions.

## Graphical abstract

This review comprehensively analyzes the energy use and demand in developing countries and developed countries, and forecasts the future energy supply and demand in combination with the past development trend.

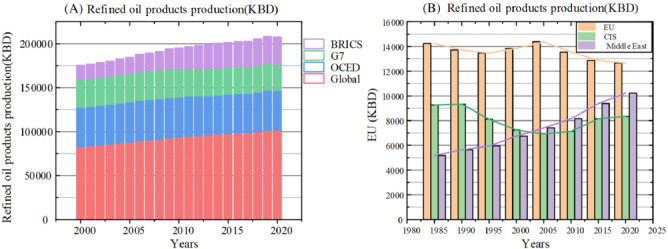

## 1 INTRODUCTION

In the seventh year after the signing of the Paris Agreement, we urgently need to make statistics on whether countries adhere to emission requirements and types of energy consumption, so as to estimate whether they can achieve the goal. In the past few years, global primary energy consumption has maintained an increasing trend, with an increase of 2.1% per year in 2017 and 2.3% per year in 2018.^1^ But as COVID‐19 swept the world, global primary energy consumption fell by 4.5% in 2020, which is the first decline in energy consumption since 2009. In addition, the proportion of fossil energy in total energy consumption increases greatly in 2020, so the decline in fossil energy consumption in 2020 is not significant.^2^ Besides, although the total energy consumption of fossil fuels shows a downward trend, the current situation that their consumption is mostly used for primary energy consumption has not changed, so further adjustments are still needed. The change in the proportion of fossil fuels can be reflected in the specific parameters of GDP, which is caused by the impact of the COVID‐19 on the development of different industries.^3^, ^4^

As the global supply chain becomes unstable under the impact of the COVID‐19, many countries are forced to face the problem of energy shortage. Whether they can stabilize their own energy supply and further maintain the stability of residential electricity consumption has become an important issue. In the past few decades, the energy industry has attracted considerable attention and is closely related to the national and social development. Energy sector plays an important role in sustainable development and wealth growth.^5^ With the COVID‐19 breaking the existing energy supply situation, some countries are forced to adjust their energy policies. This reflects the importance of grasping the domestic energy consumption. Predicting energy consumption, grasping the trend of global energy consumption and formulating appropriate energy policies will promote social development and economic construction.^6^ Energy is crucial to the achievement of global sustainable development, so it should be regarded as the top priority in both developed and developing countries. According to previous forecasts, the proportion of non‐OECD countries in the world's total energy demand will continue to increase. In 2040, it will account for 64% of the total global energy demand. Among them, Asia will achieve the greatest transformation in energy use. At present, the impact of COVID‐19 on global energy consumption continues, so energy demand will continue to be restrained. But with the expected growth of 4.6% in 2021, global energy consumption will recover to the level in 2019.^7^ On the one hand, it reflects that the impact of the COVID‐19 is shrinking. On the other hand, it also reflects that the global demand for energy has become greater than before the epidemic. Overall, with the introduction of economic stimulus plan and vaccine, the general trend of global energy consumption growth has not changed.^8^ Therefore, achieving the change in the structure of energy consumption, and improving the security of energy supply remains an urgent event.^9^ Many government agencies such as the World Energy Council (WEC), the organization of Petroleum Exporting Countries (OPEC) and the world's major oil companies or organizations have conducted a lot of investigation and prediction on energy consumption. The global crisis brought on by the epidemic has also exposed problems in today's energy system and in the relationship between supply and demand. When there are problems in energy services, some welfare services are also greatly affected. Therefore, the importance of energy services, the construction of energy systems and national energy sovereignty has become a major issue affecting social stability.^9^

Through the analysis of the information in the existing high‐quality publications, the characteristics and trends summarized from the global energy review and the change of the proportion of clean energy are analyzed.

### 1.1 Scope

**FIGURE 1:**
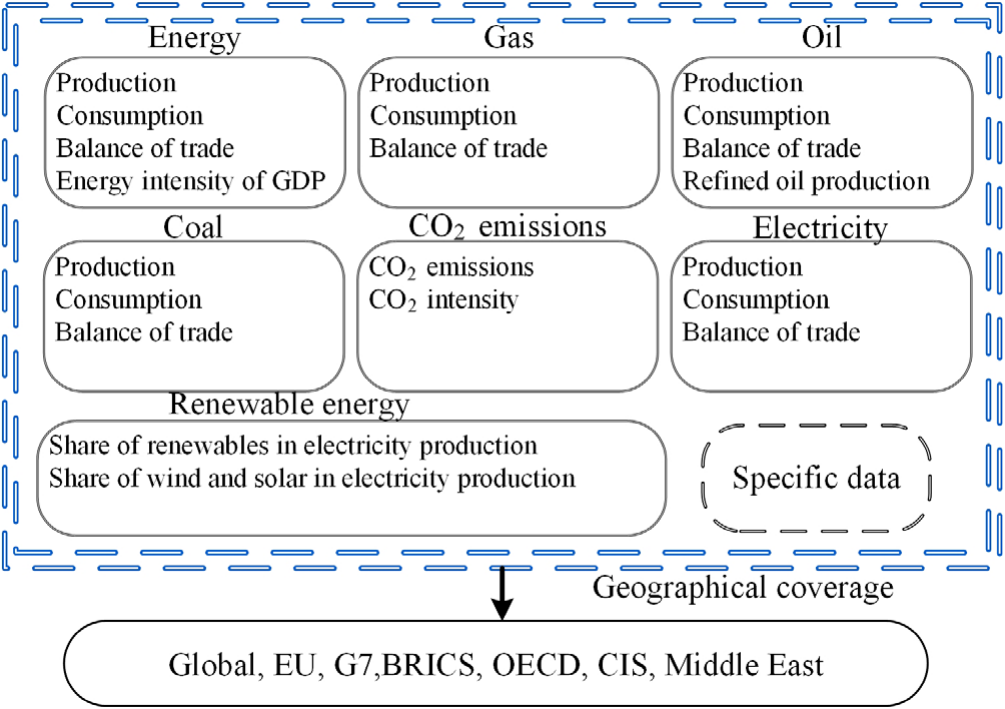
Methods of review

The research content of this paper is to predict on the time axis through the comprehensive analysis of historical energy consumption and future energy demand based on geographical scope. The geographical scope is based on OECD, G7, BRICs, European Union (EU), Commonwealth of Independent States (CIS), North America, Asia, and the Middle East. The methods of the article are shown in Figure 1.

This paper classifies energy and analyzes them by using the time series of demand and supply. By analyzing and processing different types of energy use from different perspectives, we can get a more comprehensive picture of the use of various energy sources and future trends so as to form a complete perspective and play a reference role for future policy‐making and energy structure transformation. By analyzing the available data, the overall trend of energy changes is organized to predict the future energy consumption. The conclusions obtained from these analyses can reveal the hidden dangers in the development process, so as to adjust the development direction and better grasp the impact of energy policy‐making and investment. Therefore, we can develop a clear grasp of future trends in developing countries and have a more detailed strategic reserve for policy formulation.

### 1.2 Related literature

**FIGURE 2:**
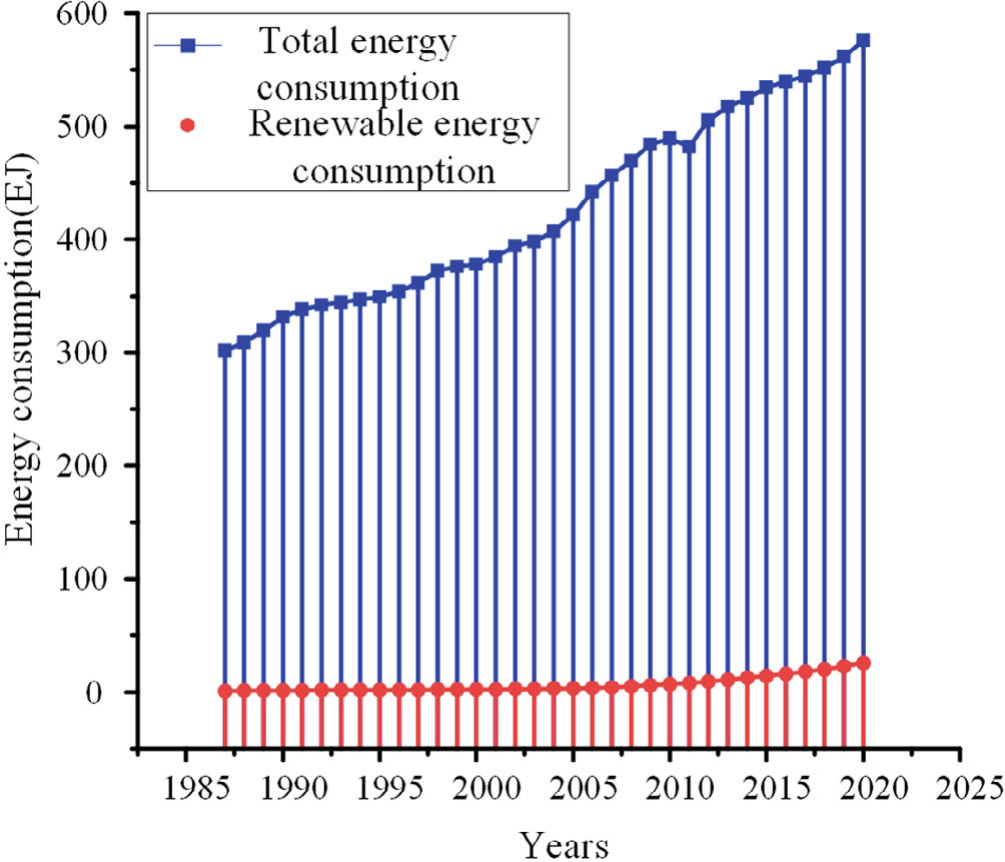
Comparison between global total energy consumption and total renewable energy consumption

The development trend and ultimate goal of energy is to achieve carbon peak and carbon neutralization, so as to reduce carbon dioxide emissions and solve the global climate change problem faced by mankind.^10^ Many international laws have been passed to limit CO_2_ emissions and set future emission targets to help the country set up follow‐up strategic policies. CO_2_ emissions will aggravate the trend of global warming, so global CO_2_ emissions should be reduced by 50% in 2050 to meet the target requirements. Although there is no clear international responsibility sharing, countries, especially several major CO_2_ emitting countries, have taken certain measures to limit CO_2_ emissions. For example, China's efforts to promote electric vehicles have reduced CO_2_ emissions from the transport sector,^11^, ^12^, ^13^ and promised at the Paris conference in 2015 that carbon dioxide emissions will not increase after 2030 and will be reduced after reaching the peak. Since 2012, through the upgrading of energy structure, the annual decline rate of CO_2_ emissions has successfully reached 1.0% from 2013 to 2016. The United States restricts CO_2_ emissions through the development of renewable energy and the reform of energy structure, and CO_2_ emissions have entered a stable downward trend. OECD countries also invest and develop renewable energy and nuclear energy.^14^ In 2050, Germany's carbon dioxide emissions are expected to be reduced to 20%. Due to the global characteristics of climate issues, optimizing the energy structure and reducing emissions are the common problems that all human citizens need to face. In order to reduce carbon dioxide emissions, we need to replace the existing energy and choose clean energy with less pollution.^15^ The comparison between clean energy and existing energy is shown in Figure 2.

At present, the development of clean energy cannot completely keep up with the growth of total energy consumption,^16^, ^17^ and its proportion is far from keeping up with that of traditional fuels. Therefore, sustainable materials, industrial symbiosis, and recycling options need to be studied. In addition, although renewable energy has many advantages,^18^ it still needs further technological development to reduce its production cost and make it profitable. Therefore, it is necessary to help start‐up of emerging energy companies through policy and the economic support.^19^ CO_2_ emissions in developing countries are mainly concentrated in transportation, agriculture, industry,^20^, ^21^ and traditional energy is concentrated in developing countries due to weak enforcement of laws and regulations. Therefore, renewable energy in developing countries still needs further development. Moreover, due to the needs of the rapid development of emerging economies, the dependence on cheap energy has also increased. It is expected that the demand for cheap energy will be in the trend of rapid development by 2025. It is very important to use greener and cleaner energy instead of fossil energy to fill the greater demand it brings. If the transformation is too slow, it will greatly increase pollution emissions. Green energy is very important for sustainable development. Green energy can protect the future environment and prevent environmental pollution caused by the use of fossil energy.

Therefore, in order to transform the energy structure smoothly and increase the proportion of renewable energy, especially in some countries with high contribution rate of traditional energy industry to economic growth,^22^ it is necessary to reduce their per capita emissions and maintain economic growth. Currently, with the improvement of renewable energy technology, it can gradually meet the energy needs of some regions, and thus greatly reduce the environmental hazards of development.

The development of renewable energy also can contribute to economic growth, promote local socio‐economic development, better protect the environment, and work with the world to curb global warming. For example, under the unified planning of the government, China has built large‐scale wind and solar power plants by reform the energy system and resource assessment.^23^ India stimulates industrial development through “green tariffs” and other relevant green policy support, and has formulated a detailed development plan for the development of solar energy.^24^ The Korean government proposed the “re 3020 plan”, which plans to make renewable energy power generation account for 20% of the total power generation by 2030.^25^ In the “The 25 years plan for national strategic emerging industries development”, China listed the renewable energy as a key strategic emerging industry to support.^26^ Through economic policy support for renewable energy and other measures,^27^ Brazil has achieved an annual growth rate of about 10.1%, achieved 89% of total power generation from renewable energy sources, and has formulated a sustainable development plan. Wind energy and other green energies have developed at a high speed. European countries have also replaced the energy structure from the aspects of energy conservation,^28^ energy efficiency and renewable energy replacing fossil fuels, so as to reduce the consumption of fossil energy by supporting the development of research projects.^29^ Through the promotion of electric vehicle technology, the EU region has achieved the penetration of renewable energy in the field of transportation, and set a target of 32% of renewable energy in 2030. Ireland has increased the share of renewable energy on the grid to 75% and plans to increase the share of renewable energy to 95% by 2030.^30^ OECD countries and other developed regions realize that in addition to the level of technology, the energy development of enterprises and governments is to some extent based on the environmental awareness of people,^31^ and through speech education and other activities, citizens' awareness of environmental protection has been improved.^32^, ^33^ These actions have successfully improved people's support for the environmental protection industry, and created an environment suitable for the development of renewable energy industry, and thus accelerating the development of clean energy technologies.

In addition to the industry and transportation that people often talk about, the building sector also accounts for a large amount of energy consumption.^34^, ^35^, ^36^, ^37^ The building sector is a major energy‐consuming sector and is estimated to account for more than a third of the world's energy consumption.^38^, ^39^ At present, the energy consumption of buildings has been reported to account for 67% of the energy demand worldwide.^40^ The energy consumption of buildings can be traced back to the different types of energy used by power stations in different regions.^41^, ^42^ Therefore, Adjusting the energy used in its mainstream can help the construction industry to become more environmentally friendly.^39^, ^43^, ^44^, ^45^, ^46^

Although most people in the world have adapted to the ubiquitous power supply, it cannot be ignored that about 2 out of 10 people in the world still live in the areas without electricity.^47^ According to the definition of IEA, 126.7 million people did not have access to electricity in 2010. This figure increased to 128.5 million in 2012, meaning that population without power supply growth exceeded the number of new power connections. More than 95% of people without electricity live in developing regions, in sub‐Saharan Africa, Asia, and Latin America.^48^ By studying the economic growth, industrial development and supply relationship of countries in underdeveloped areas,^49^ this paper studies their energy policy and development driving force. At present, due to the climate problems caused by CO_2_ emissions, if we want to meet the power needs of more people while transforming the global energy society, we need to develop renewable energy to a greater extent. By 2019, renewable energy has provided more than 27% of the increased power generation. Through the development of renewable energy, we can help the living standards of developing regions continue to grow and access clean water, convenient power.^50^, ^51^ For example, in Iran, rural electrification has been promoted through the use of renewable energy and fuel power generation, and the rural electrical network has been improved through the use of renewable energy such as photovoltaic panels and wind turbines, combined with battery packs.^52^ In South Asia, through the use of ICT trade, some economies in South Asia have received economic support, so that they can carry out energy transformation and promote the development of renewable energy technology. These technologies have broken trade barriers and promoted the development of energy technologies, thus making the power supply of these economies more stable. At the same time, they have solved the problem of low rural electrification rate in South Asia.^53^ Such research can help the government formulate more reasonable energy policies and help the country realize the transformation of energy structure, so as to achieve sustainable development in the long term.^54^ During this epidemic, the energy industry has been under great pressure. When the existing planning is impacted, the proportion of more stable fossil fuels and other traditional energy in the energy system has been increased. This is due to further adjustments made by the government to ensure the proper functioning of the society when there are challenges in energy supply and demand. These measures would further contribute to the increased dependence on conventional fuels and slowing of CO_2_ emission reductions. Therefore, for some countries, which rely heavily on traditional fuels, it is particularly important to fully prepare the strategic system to deal with the impact of the epidemic by adjusting the development strategy of renewable energy. Therefore, the research and statistics on past development and strategies can help these countries build a strategic system, so as to smoothly carry out their own transformation under the challenge of the epidemic. In order to ensure power supply, the improvement of energy efficiency is also an urgent problem to be solved. Developing renewable energy and improving the stability of energy supply in developing countries can reduce energy poverty, so as to ensure the stability and convenience of power supply. The development of renewable energy can enable developing countries to expand their power supply with lower carbon. Therefore, by analyzing the transformation and development of their energy structure, we can better formulate relevant energy policies. This allows for better energy restructuring in emergency situations.

### 1.3 Article structure

Based on the division of regions, this paper will analyze different types of energy consumption, and try to predict the trend of energy consumption and future consumption. The rest of the paper will be divided according to the following structure. The second part will make statistics and analysis on the historical consumption of different types of energy, and list the change trend of carbon dioxide emissions. The third part will comprehensively analyze and try to analyze and predict the future energy consumption trend. The fourth part will summarize this study.

## 2 GENERAL TREND OF GLOBAL ENERGY CONSUMPTION

In this part, the historical energy consumption and past trend will be analyzed, and different kinds of energy will be described from different perspectives.

### 2.1 Energy trends

With the development and progress of mankind, the energy used by mankind also shows different characteristics in different times. With the further development of human society, the energy currently used also needs to be changed in the next step. As countries put forward their own energy transformation plans, energy transformation and carbon dioxide emission reduction projects are being carried out all over the world. The key question of energy consumption research is, how will global energy consumption develop in the future? To what extent will energy technology develop by then? By reviewing the energy policies, latest technology trends and data of various countries, we can correlate energy consumption with GDP, so as to obtain a clear energy change trend.^55^ Through the analysis, it can be seen that energy consumption is being reduced and energy transformation is being carried out all over the world. To study the current situation of energy transformation in various countries, it is necessary to systematically analyze the country from the policy and other levels, such as formulating long‐term sustainability goals, implementing emission reduction requirements, and providing market support for new energy and new technologies. At present, all regions have made certain requirements and efforts for energy conservation and emission reduction,^56^ and have achieved quite remarkable results. For example, in Germany, the energy consumption of residents decreased by 8% from 1990 to 2014,^57^, ^58^ during which other parts of Europe also declined to varying degrees, by helping emerging countries start renewable energy, the EU has successfully encouraged them to further development and promoted the development of renewable energy.^59^ In Asia, some changes have also been made, such as India has set a target of 175 million kilowatts of renewable energy by 2022.^60^

#### 2.1.1 Production and consumption balance

In order to have a more specific analysis and data on the energy consumption and demand of various countries, it is necessary to make a clear estimation of the energy consumption and demand by using the primary power generation. In order to further analyze the trade balance of different types of energy, other segments of energy trade, such as foreign trade, are classified. The data in the table below can be used to arrange the analysis of the trade balance and the supply and demand balance for different countries and regions.

**FIGURE 3:**
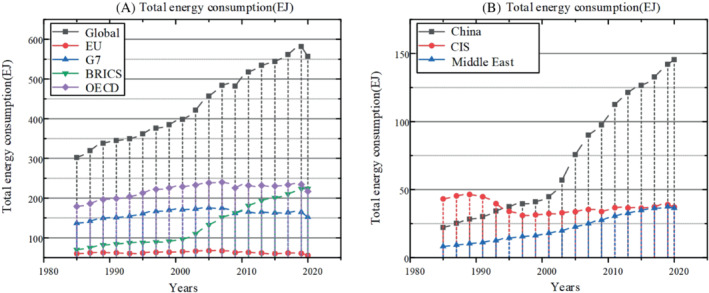
(A) Total energy consumption of the Global, EU, G7, BRICS, OECD, (B) Total energy consumption of the China, CIS, and Middle East.

**FIGURE 4:**
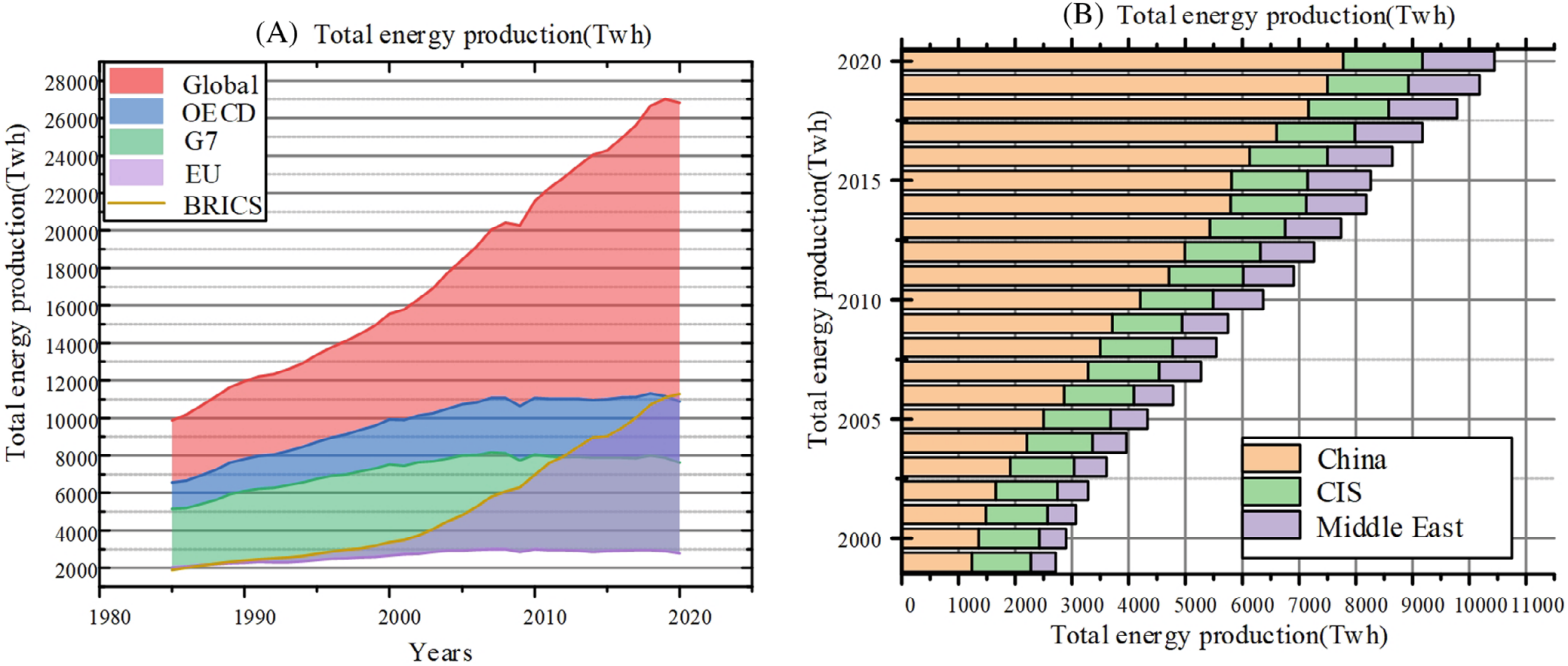
(A) Total energy production of the Global, EU, G7, BRICS, OECD, (B) Total energy production of the China, CIS, and Middle East

As shown in Figure 3, we can see the total energy consumption of countries sorted by time axis. In the graph above, we can see the growing trend of energy demand and the huge growth of consumption in some countries such as China in recent decades. Thus, we can see that the balance between energy demand and manufacturing is not a simple dynamic balance, but shows complex changes with the impact of national economic growth requirements and changes in the international environment. And as can be seen from the Figure 4 below, the relationship between energy supply and demand in different regions and countries has changed greatly in the past few decades. First of all, except for the impact of the epidemic in 2020, energy consumption has maintained a growth relationship. According to the IEA's 2021 Energy Yearbook, the growth trend has already got rid of the impact of the epidemic. In addition, the energy production of various countries has also maintained rapid growth. For example, Asian countries have experienced a huge growth, far surpassing the United States as the largest energy supplier. Apart from the reasons for their rapid development, Asian countries also bear more primary energy consumption, which has a certain problem of low energy utilization.^61^ While some developed regions put forward emission reduction targets, more fossil fuel consumption and high emission industries are concentrated in Asia, which is also one of the reasons for the rapid growth of energy demand and supply. Figure 4A,B shown the energy production in different regions. According to the data, the total energy output of OECD changes little, while the production of BRICs countries has always been in high‐speed growth.^62^ In addition, it can be seen from the figure that the energy demand has increased rapidly in the past decade, and with the rapid growth of energy demand in Asia, the transformation of energy has become urgent. Therefore, in order to achieve rapid transformation, the government needs to introduce renewable energy investment and encourage the use of clean energy, so as to maintain the downward trend of greenhouse gas emissions and focus economic growth on the development of renewable energy.^63^ It can be seen from these figures that in recent 10 years, with the rapid development of various countries, energy production, and consumption have been both increasing, which can be related to the trend of economic growth. In some countries, there is no positive correlation between energy consumption and production. For example, in some periods, energy production decreases, but at the same time, energy demand will increase. Overall, the main growth comes from the developing countries such as China, while energy consumption in developed regions has stabilized.^64^, ^65^

#### 2.1.2 Relationship between energy and GDP

The trade surplus can be obtained through the statistics and comparison of the import and export trade value. By analyzing the trade surplus and the changes of GDP in the corresponding period, we can analyze the relationship between them. In addition, the relationship between the energy consumption in this region and the change of GDP in the corresponding period can be obtained. Here, energy intensity is uniformly defined as: total energy consumption/total GDP.

(1) Energy intensity=Energy consumption/TotalGDP

**FIGURE 5:**
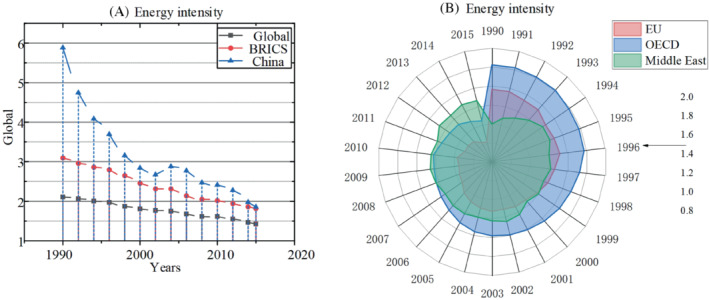
(A) Energy intensity of the Global, BRICS, China, (B) Energy intensity of the EU, OECD, and Middle East

The main way to reduce energy intensity is to develop renewable energy, but when the formulation of structural adjustment strategy is wrong, the development of renewable energy will have a certain negative impact. Overall, in general, the development of renewable energy is far higher for the economic growth of developing countries than that of developed countries.^66^, ^67^ It is estimated that by 2023, the share of renewable energy consumption will reach 12.4%. Therefore, developing regions need to conduct a comprehensive analysis of past investment experience, so as to reduce investment costs and use renewable energy to promote their own economy. Some energy supply countries also have similar problems. For example, Saudi Arabia has excessive energy use and low fuel prices, so it has a very low energy utilization rate. Therefore, although it is in developed areas, it still has high‐energy intensity due to this situation. In addition, the overall global energy intensity has been in a downward trend.^68^ Due to the slight rise of the COVID‐19 in 2021, there is still a large gap from the goal of net emissions in 2050. As shown in Figure 5, the energy intensity of developed regions is controlled below 1.5 KWh per $, which is still no less than 2 KWh per $ for most Asian regions, implying that Asia accounts for more primary energy consumption. This is an issue that must be taken seriously.

#### 2.1.3 Energy trade

With economic development, developed countries have shown a certain dependence on developing countries in many aspects. In addition to some consumer goods and factories, energy in some developing countries is relatively cheaper. Therefore, the energy market has become more global, and some countries have become dependent on the global energy supply chain to maintain their energy stability, especially some developed countries with relatively scarce resources. Once the international trade or supply chain fluctuates, it will also have a certain impact on the stable supply of domestic energy.^69^ This effect is particularly obvious during the epidemic period. Heating and power supply in many countries are unstable due to energy shortage, which has a negative impact on social stability.

The scale of the world energy market is affected by economic globalization and continues to expand with the development of transportation technology. In the 1960s, most countries were able to maintain the balance of energy import and export, and only a few poorer countries exported much more than imported due to their economic dependence on energy trades. During this period, with the improvement of energy technology and the expansion of trade scale, more and more developing countries have achieved rapid economic growth through energy sales. Until 1990, developing countries played the role of exporters in the market. With the reduction of their dependence on energy trade for economic development, the balance between energy import and export of most countries was stabilizing. This trend is also reflected in the decline in energy GDP intensity. For example, in order to resist the impact of the global economic crisis in 2008, China significantly increased its integration into the global market in 2010 through the 400 million yuan stimulus plan.^70^ During this period, China made its energy import and export trade grow exponentially by adjusting tariffs and the policy of import and export. Renewable energy has developed in Africa in recent decades, and the export volume began to increase rapidly after meeting domestic demand in 2000, which has brought considerable economic income to the region.^71^, ^72^ With the further development of science and technology, advanced transaction modes such as P2P business model appear, which further reduces the cost of energy trade. By analyzing the balance of energy supply and demand and the changes in the amount of import and export, decision makers can use energy storage, change supply plans, and economic stimulus to achieve long‐term evaluation. While ensuring the balance between supply and demand, we can increase domestic income through energy exports.

### 2.2 Commercial trade, production and refining of oil

As one of the most popular energy sources used by mankind, oil has its applications in all fields of human life.^73^ As a result, it is not possible to fully measure the use of primary and secondary power. The significance of oil to mankind is not just an energy, but as one of the most valuable commodities in the world. In some countries, oil trade has even become a major source of income and growth for the economy. Oil not only brings considerable wealth to these countries, but also establishes a considerable number of jobs and development opportunities for these countries. Some Middle Eastern countries derive 80% of their economy from oil exports.^74^ Therefore, it is also necessary to measure each step of oil production and trade. In this section, the trade and production of oil will be further detailed.

#### 2.2.1 Oil trade and balance trends

**FIGURE 6:**
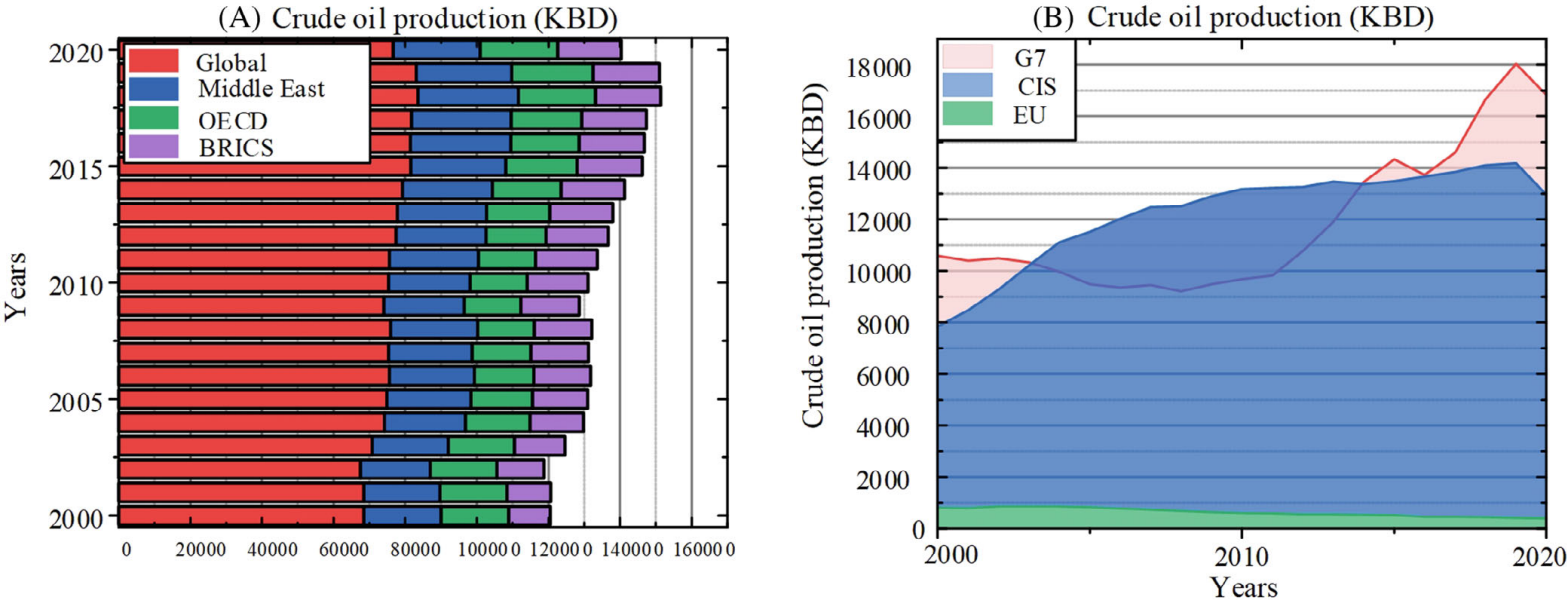
(A) Crude oil production of the Global, Middle East, OECD, and BRICS. (B) Crude oil production of the G7, CIS, EU.

Oil trade refers to all kinds of liquid hydrocarbons without purification process. After refining, oil can be divided into a variety of raw materials, plastics, nylon, and other substances. The oil exploitation volume of the United States ranks first in the world, and its use volume also ranks first in the world. During the period when the United States announced the launch of the Paris Agreement, its oil trade gained higher activity. North America and the Middle East account for 56.5% of the world's oil production.^75^ The specific crude oil production is shown in the Figure 6A,B below. Saudi Arabia followed, but Saudi Arabia accounts for only 0.66% of global consumption. Japan has become the second largest crude oil consumer in the world. In addition, affected by the epidemic, the demand for crude oil experienced the largest sharp decline in 2020, with global production falling by about 7.7%.^76^ However, with the recovery of trade in 2021, it has returned to the level of 2019, so the demand for crude oil is expected to remain in an increasing trend in the future. Except for the fluctuations in 2020, the oil consumption in all regions of the world is growing steadily, there is no sudden increase or decrease, and the change rate tends to be stable. According to the forecast, as countries are considering environmental protection, the oil trade volume will enter a gradual downward trend.

#### 2.2.2 Oil consumption

**FIGURE 7:**
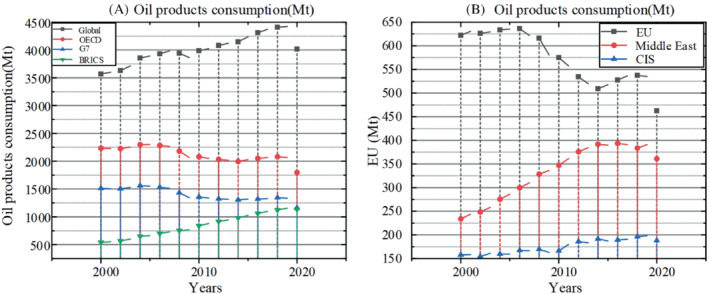
(A) Oil products consumption of the Global, OECD, G7, and BRICS. (B) Oil products consumption of the EU, Middle East, and CIS.

The actual oil consumption can be obtained by counting the number of oil put into refineries in different countries and regions. As shown in Figure 7A, consumption in the BRICs countries has rapidly approached that in G7 countries. Affected by the epidemic, the consumption in different regions of the world decreased in 2020, but it still maintained an upward trend compared with 2009. Consumption in North America, the European Union and other countries changed slightly,^77^ while that in India, China and other countries increased rapidly. With the efforts of energy transformation, U.S. oil consumption has decreased significantly. Therefore, Asian countries need to make a series of policies to ensure that the economy continues to grow at a high speed and reduce per capita oil consumption. The specific change trend is shown in the Figure 7A,B.

#### 2.2.3 Refined oil production

**FIGURE 8:**
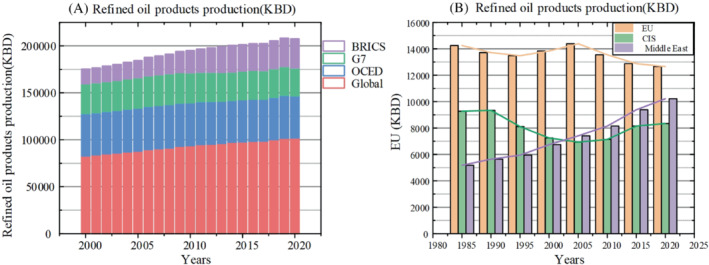
(A) Refined oil products production of the Global, G7, OECD. (B) Refined oil products production of the Middle East, CIS, EU.

Affected by the epidemic, the global total oil production in 2020 showed the largest decline in history. As shown in the Figure 8, BRICs countries once again show growth that cannot be ignored. Asia's oil consumption is still high, but its oil production growth is slow, and it has maintained a large amount of oil imports for a long time.^78^ The total global oil production in 2020 was 41.651 million tons, which was lower than 42.272 million tons in 2014, this shows that the epidemic has brought a huge impact. OECD, BRICs, G7, and the United States are still the main producers. With the rapid development of BRICs, oil production has exceeded that of OECD countries.

### 2.3 Natural gas

Compared with other fossil fuels, natural gas is considered to be a cleaner energy.^79^, ^80^ From fossil fuels to natural gas power generation has become the mainstream of low‐carbon emission liberation.^81^ Most countries have begun to adopt this method to reduce carbon emissions while maintaining normal power and industrial life. The use of natural gas in power and industrial sectors accounts for more than 80% of the global total demand.^82^

#### 2.3.1 Production

**FIGURE 9:**
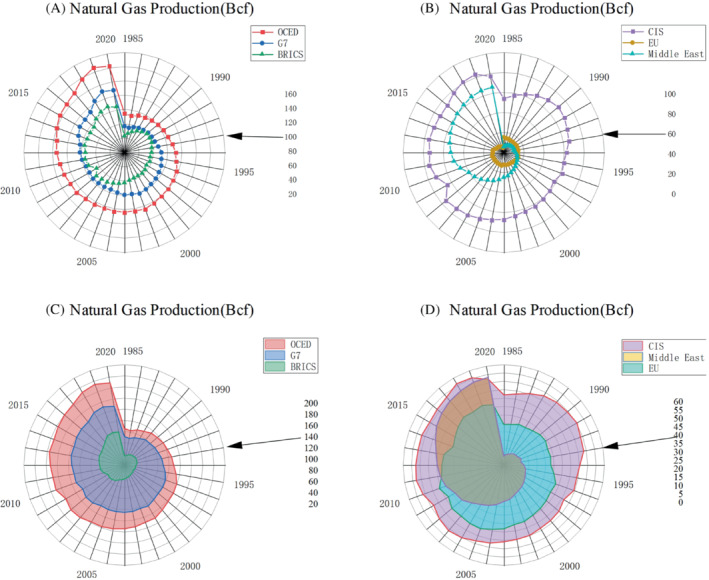
(A) Natural Gas consumption of the OECD, G7, BRICS, (B) Natural Gas consumption of the CIS, EU, and Middle East. (C) Natural Gas production of the OECD, G7, BRICS, (D) Natural Gas production of the CIS, EU, and Middle East.

It is estimated that the global natural gas demand will maintain an annual growth rate of 1.9%.Figure 9A,B shows global natural gas production. The production of countries in the Middle East and Asia has increased rapidly as shown in Figure 6B. In 1970, the output of Asia was only 1.46 BCF, but it has increased to 62.92 BCF in 2020. Middle East countries still have abundant natural gas reserves, and their consumption is expected to be in a sustained growth stage. With the popularization of natural gas use, it is expected that the annual growth rate of each region will be in the rising stage in the future.

#### 2.3.2 Consumption

The United States has the largest total consumption in the world, with a total consumption of 80.28 BCF in 2020. It is expected that by 2040, the consumption of natural gas in the United States will reach more than 40%, surpassing oil as the main energy. China's consumption has increased rapidly and has reached the second place in the world. Through the use of natural gas, China has reduced its carbon emissions by 30%. Figure 9C,D shows global natural gas consumption. This trend reflects that a considerable number of countries have noticed the low‐carbon advantages of natural gas. Since 2015, natural gas consumption has entered a stage of rapid development and is expected to maintain rapid development.

### 2.4 Coal consumption and major dependent countries

**FIGURE 10:**
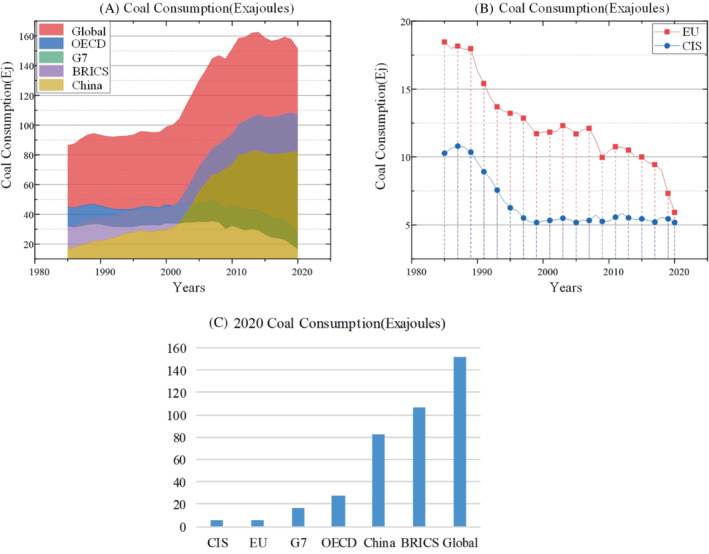
(A) Coal consumption of the Global, OECD, G7, BRICS, China, (B) Coal consumption of the EU, CIS. (C) 2020 Coal consumption of the Global, OECD, G7, BRICS, China, EU, CIS.

As one of the longest used energy sources, coal still has a large consumption. But the United States, the European Union and other countries are already working to reduce the use of coal in power generation, and the major consumption of coal is concentrated in Asian countries. Figure 10A,B shows global natural gas consumption. Among them, China is the country with the largest dependence on coal in the world.^83^, ^84^ In 2020, China's coal consumption accounted for 54.33% of the world and 56.55% of China's total fuel consumption,^85^ as shown in the Figure 10C. As one of the most populous developing countries, India's coal consumption is also high. But with the adjustment of the government's energy consumption policy, India's coal consumption has decreased on a small scale in 2020. However, with the impact of the COVID‐19, it is expected that the global coal consumption will experience a large‐scale increase in 2021.^86^ From 2014 to 2020, coal consumption has always been in a downward trend. Among them, the consumption of OECD in 2020 decreased by 15.2%. In 2020, EU countries decreased by 19.4%. In 2020, the coal production in Asia was 58.794 million tons, accounting for 75.9% of the total global coal production. Moreover, with the predicted energy demand continuing to rise, Asian countries will still have an upward trend in coal consumption due to their dependence on coal for power generation.

### 2.5 Renewable energy

To face with the increasingly severe global climate problems, it is imperative to reduce environmental damage caused by human development while maintaining stable GDP growth. The use of green renewable energy can solve this problem well, but due to the lack of effective development and promotion, the development of renewable energy is by no means an overnight problem. Due to the uneven distribution of fossil fuels among countries, the supply stability of some resource deficient countries depends on other countries, which brings a series of political and other problems.^87^ As fossil fuel reserves decline, the difficulty of collecting fossil fuels will also rise, which will inevitably bring about an increase in prices. Therefore, the development of renewable energy has become an urgent problem in many countries. Renewable energy has developed rapidly in recent decades, and both fuel consumption and power generation are on the rise according to energy transition plans formulated by countries.^88^

#### 2.5.1 Renewable energy share of total power generation

**FIGURE 11:**
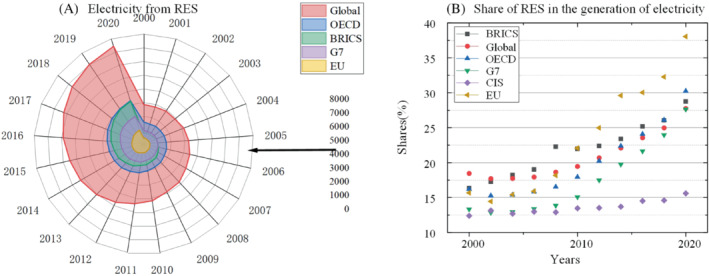
(A) Electricity from renewables of the Global, OECD, BRICS, G7, and EU. (B) Proportion of renewable energy power generation in total power generation of the Global, BRICS, OECD, G7, CIS, and EU.

With the development of renewable energy technology, the efficiency of power generation using renewable energy has been improved, so the power generation has increased greatly.^89^ Figure 11A shows electricity generation from renewable energy sources.^90^ Figure 11B shows proportion of renewable energy power generation in total power generation. As can be seen in this figure, the global renewable energy power generation accounted for 28% of the total power generation in 2021, which decreased slightly compared with 29% in 2019. This is due to the impact of the epidemic, and the use of fossil fuels increased again. However, as the impact of the COVID‐19 subsides, the share of renewable energy in power generation will continue to increase, and it is expected to reach 84% of the total power generation in 2040. Renewable energy has become the mainstream power generation in some developed regions. In 2021, only 0.46% of Norway's electricity came from fossil fuels. Renewable power generation in Brazil accounts for 79% of total power generation and 69% in Canada.^91^ Although China's renewable energy power generation accounts for a large proportion of the global total, accounting for 30% of the global total in 2020, China's renewable energy power generation accounted for only 26% of the total power generation in 2021.^92^

#### 2.5.2 Share of solar and wind power generation

With the promotion of renewable energy in various countries, renewable energy has made great progress in recent years. In terms of solar energy, China's loading capacity accounted for 35.9% of the world in 2020, and it is expected to grow in the future with the further support of the government. In terms of wind energy, China still ranked first, followed by the United States. Despite the economic downturn caused by the impact of the COVID‐19, the development speed of wind energy and solar energy has still reached the highest point in recent years. It is expected that with the reduction of the impact of the COVID‐19, there will be a great leap forward in development. As a global leader in renewable energy power generation, China's installed capacity of solar and wind energy is expected to reach more than 1200GW in 2030.

### 2.6 Production and use of electricity

In this part, the power generation demand can be analyzed and predicted in detail by analyzing the total power generation and the part occupied by domestic power consumption.

#### 2.6.1 Production

**FIGURE 12:**
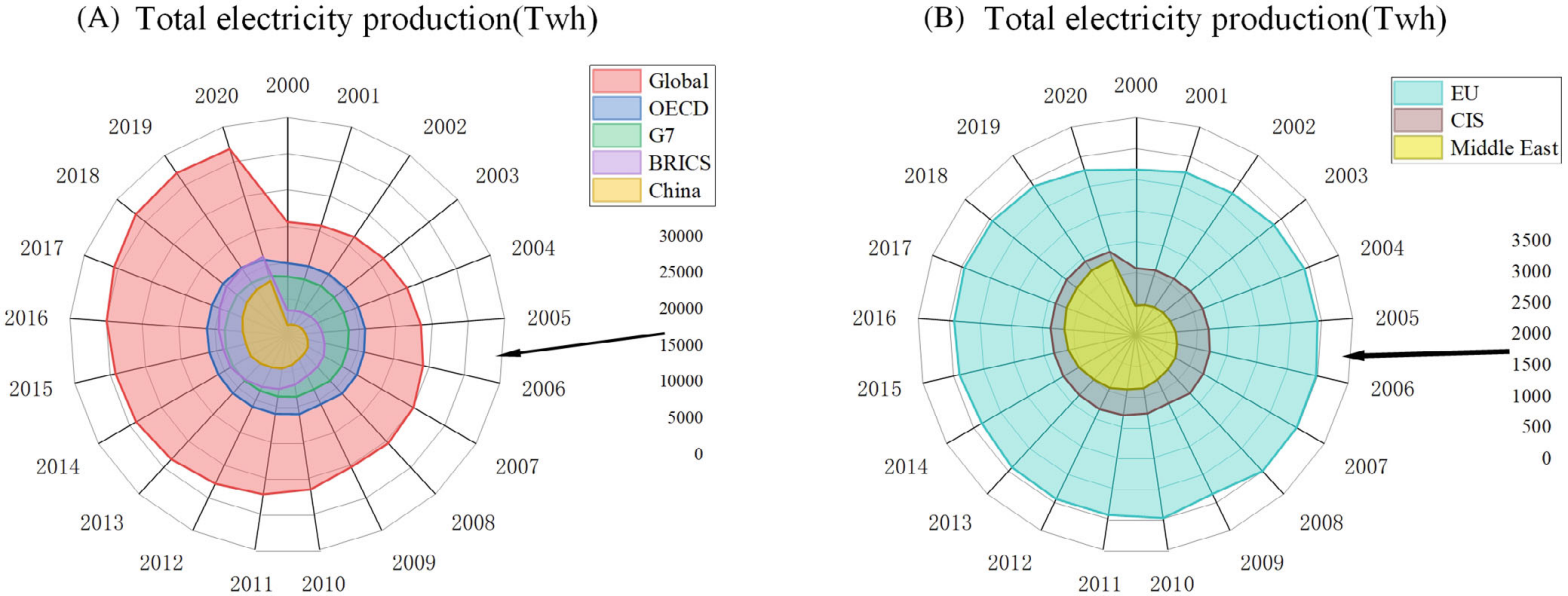
(A) Total electricity production of the Global, OECD, G7, BRICS, China. (B) Total electricity production of the EU, CIS, and Middle East.

Figure 12A,B below shows the world's total power generation. It can be seen that with the rapid development of Asian economy, Asian countries have accounted for nearly half of the global power generation since 2010. With the development of Asia,^4^ the power generation in Asia is still in a steady rising trend. Power generation growth in developed countries has entered a moderate phase. Since 2005, the power generation in the United States has entered a slow growth in 2005, the total power generation in the United States was 4322.8 TWh and then it reached 4411.2 TWh in 2019, with an increment of only about 2%. During this period, the power generation in the European Union declined by 2%. Meanwhile, Asian countries have entered a period of rapid development over the same period,^93^ with an increase of 97.3%, nearly doubled. In 2020, under the impact of the COVID‐19, the power generation of OECD and the European Union was in a downward trend, but some emerging economies, such as China, still maintained the momentum of growth. China's total power generation in 2020 was 7778.76 TWh, an increase of about 3.3% compared with 2019. Global electricity generation in 2020 fell for the only time in decades, with a total power generation of 26823.24 TWh, 27000.95 TWh in 2019 and a decrease of about 0.9% in 2020. However, with the economic recovery, it is expected to usher in a rebound and rise in 2021.

#### 2.6.2 Domestic electricity consumption

From 2009 to 2019, the overall power consumption of North America and EU has stabilized, with an annual growth rate of less than 0.5%. Compared with the rapid development of BRICs. By 2020, electricity consumption in BRICS countries has surpassed that of OECD countries, and the growth rate is still fast. Total global electricity consumption fell in 2019 and 2020 for the only time in decades as a result of the COVID‐19 pandemic. With the economic recovery, countries in rapid development, such as India and China, saw a certain increase in power generation in the fourth quarter.^94^ In recent 10 years, power consumption has been growing, especially in the rapidly developing BRICs countries. In 2020, China's total power consumption has accounted for about 30% of the world, and with its rapid development, this proportion will continue to increase.

### 2.7 CO_2_ emissions

In 2020, global CO_2_ emissions show a downward trend for the first time, dropping 6.2% from last year.^95^ This is because with the signing of the Paris Agreement, countries promulgated their own emission reduction targets, to reduce carbon dioxide emissions have made certain efforts.^96^

#### 2.7.1 Emissions

**FIGURE 13:**
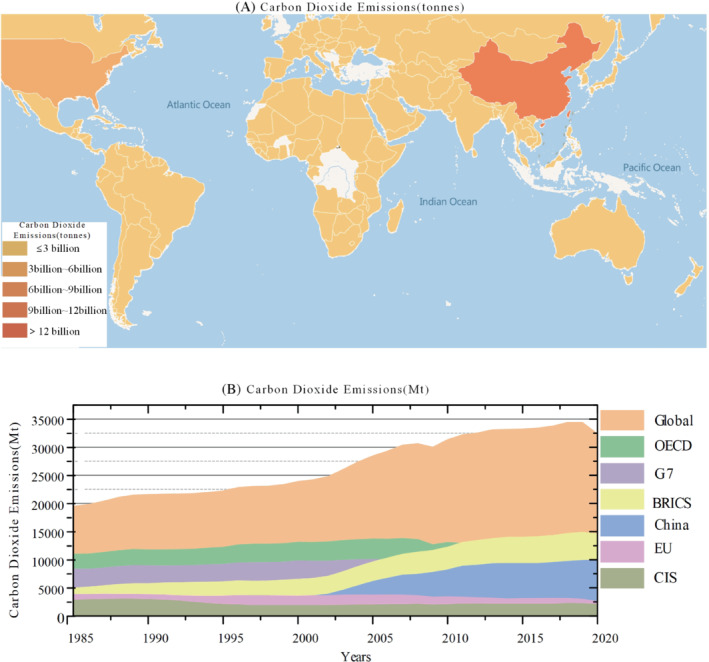
(A) Worldwide carbon dioxide emissions (tonnes), (B) Carbon dioxide emissions (million tons) of Global, OECD, G7, BRICS, China EU, and CIS

The carbon emissions of different regions are shown in the Figure 13A below through the thermal map, which can more intuitively see that China's carbon emissions are already in the first echelon. With its rapid economic development, China's carbon dioxide emissions have been growing,^97^ with an average annual growth rate of 2.35% as shown in Figure 13B. In 2006, China overtook the United States to become the world's largest emitter of CO_2_. China is the only country with rising CO_2_ emissions in 2022.^98^ Although China has issued quite strict policies to control the carbon emissions of domestic enterprises, due to the different degree of restrictions of environmental rules in different regions, there are carbon intensive industries in some regions.^99^, ^100^ Therefore, China still needs more work to reduce CO_2_ emissions. As the second largest carbon dioxide emitter, the United States began to decline year by year after reaching its peak in 2007, and decreased by 11.4% by 2020, making its own contribution to reducing greenhouse gas emissions. In addition, as the world's second fastest growing economy, India has taken a series of energy measures in recent years, reducing carbon dioxide emissions by 7.1% in 2020. Carbon dioxide emissions in the EU and other economies have declined to some extent.^101^ The EU promises to reduce its CO_2_ emissions by 40% by 2030. With the current trend, the EU will be able to achieve this goal. In general, all countries have made certain efforts to reduce carbon dioxide emissions.

#### 2.7.2 Carbon intensity

Every country is considering a smooth energy transition and reduction of domestic carbon emissions while maintaining current economic development dynamics. Therefore, if we want to achieve the goal of reducing global carbon emissions by 2030, and further improve the climate problem, we must balance the relationship between CO_2_ emissions and GDP,^102^ we must address the “carbon intensity” of our economy. Carbon intensity is calculated as CO_2_ emissions per unit of GDP, this means that the implementation of low carbon production methods and the promotion of low carbon emission energy can reduce carbon intensity.

Overall, in the past decade, the global carbon intensity has always been in a stable downward trend. In 1960, the global carbon intensity was 0.71 kg/$, while in 2018, this figure has decreased to 0.32 kg/$. This downward trend is mainly due to the energy transition in developed countries, which has led to a steady decline in carbon intensity. Since the 1990s, the carbon intensity of these countries began to decline at a high speed. The United States decreased from 0.54 to 0.32 kg/$, and European countries decreased from 0.58 to 0.28 kg/$.

The carbon intensity of some developing countries is also closely related to their own development process. For example, China's carbon intensity reached a peak of 1.13 kg/$ in 1960, and then decreased to 0.49 kg/$ with industrial technology development in 1967. But with its rapid development, the carbon intensity stepped into the rising stage again from 1967 to 1978, reaching 0.90 kg/$. In recent years, with China's efforts in energy transformation and industrial structure, China's carbon intensity has also begun to decline steadily, and has entered a downward trend, falling to 0.57 kg/$ in 2018.^103^ With the further implementation of industrial reform in China,^104^ it will still be in a downward trend in the future. However, China's carbon intensity is distributed from east to west, and advanced enterprises in the coastal and inland areas are unevenly distributed. The existence of carbon‐intensive ports, is also not conducive to the further reduction of carbon intensity. Therefore, it is necessary to further restructure the final demand in the central and western provinces in order to better meet the carbon intensity target for 2030.

## 3 ENERGY DEMAND AND EMISSION FORECAST

In this section, we can refer to the previous data to roughly grasp the future trend. Although the relationship between world energy consumption and demand fluctuates today, projections of future energy demand and supply will remain constant as we approach 2030,^105^ and there are barely high impact changes in national policies so far.^106^

Tables 1 and 2 show the projections from 2022 to 2040. The specific results of the projections are summarized by calculating the annual rate of change of the data of recent decades and extracting their trends, which leads to a projection of the total consumption in 2040. Global energy consumption should continue to grow at a rate of about 1.8%. As the impact of COVID‐19 wanes, energy consumption in 2021 will rebound to the 19‐year level and continue to grow at a high rate. As a result, global economic consumption should reach about 795.95 EJ by 2040. Asia will continue to lead in energy consumption with a growth rate of 3.2%, followed by the Middle East with 3.0% and Africa with 2.5%. In contrast, the EU region will remain relatively stable, declining at a rate of 0.2% per year. The North American region also has a low growth rate of about 0.5%. Thus, it is estimated that by 2040, energy consumption in North America, EU, Asia and the OECD will be 10,906 GJ, 5230 GJ, 40,124 GJ and 28,012 GJ, respectively. It can be seen that the growth in energy consumption comes mainly from Asia, while energy consumption in other regions tends to stabilize. This is due to the concentration of energy companies set up in the Asian region in recent years, as well as the Asian countries introduced policies to maintain the growth of the energy economy.^107^

**TABLE 1:** Total primary energy consumption requirement

Category	Unit	2020	2023	2024	2025	2030	2035	2040
World	EJ	557.10	562.67	563.80	564.92	621.42	622.66	623.90
EU	EJ	55.74	54.62	53.53	52.46	52.41	52.35	52.30
G7	EJ	152.03	152.52	152.02	151.54	151.05	150.56	150.08
BRICS	EJ	224.25	231.46	238.92	246.61	325.99	336.49	347.32
North America	EJ	107.90	107.96	108.03	108.09	108.42	108.74	109.07
Asia	EJ	253.72	262.09	262.95	263.82	303.39	348.90	401.24
OECD	EJ	217.11	218.03	218.95	219.87	246.67	249.68	280.12

**TABLE 2:** Final demand for electricity consumption

Category	Unit	2020	2023	2024	2025	2030	2035	2040
World	TWh	26823.25	27604.80	28409.13	29236.90	32427.73	35966.80	39892.12
EU	TWh	2770.56	2792.34	2814.29	2836.41	2938.13	2961.23	2984.51
G7	TWh	7627.52	7644.20	7660.92	7677.67	7763.56	7850.41	7938.23
BRICS	TWh	11284.99	11688.53	12106.50	12539.43	14492.56	15010.81	15547.58
North America	TWh	5243.64	5313.11	5383.51	5454.84	5799.85	5876.70	5954.56
Asia	TWh	14124.62	14937.07	15796.25	16704.84	19503.24	20625.06	21811.41
OECD	TWh	10880.82	11036.02	11193.42	11912.75	12082.66	12254.99	12429.78

In terms of electricity consumption, future electricity demand can be predicted by the amount of electricity generated in recent decades. According to the forecast, in 2040, Asia will account for a larger portion of total electricity generation due to the rapid growth rate of Asian economies. By 2040, global electricity generation is expected to reach 39,892.12 TWh and Asia will reach 21,811.41 TWh, accounting for a large share of the total. The energy consumption in other regions is 2984.51 TWH in EU, 5954.56 TWH in North America, and 12429.78 TWH in OECD. it can be seen that the focus of world electricity production has shifted to Asia.^108^ In addition, in terms of energy structure transformation, the share of sustainable energy can be obtained by calculating the proportion of sustainable energy in the total energy consumption.^109^ Currently, the most used renewable energy sources are solar and wind. All regions of the world have made good development, and the proportion of sustainable energy is still increasing with the support of national policies. By calculating the proportion of electricity generated from renewable energy sources and then calculating the annual rate of change, it can be predicted that the proportion of electricity generated from renewable energy sources in 2040. According to the calculation, by 2040, Asia will rank first in the world in terms of installed capacity, but will still be second to the U.S. in terms of power generation as a percentage of total power generation.^110^ As for natural gas resource consumption, the United States will still account for the largest share, although it is declining year by year. China will account for 18.55% of total consumption, while OECD countries will account for 39.33%.^111^

In addition, with the energy transition and the increase in the proportion of renewable energy, CO_2_ emissions will show a steady downward trend. As shown in Table 3, by 2040, total global CO_2_ emissions are expected to decline by about 30%.^112^ This result is obtained by taking into account the annual decline rate of CO_2_ emissions in each country in the last decade and the newly introduced energy policies of each country, so as to grasp the rate and trend of decline and obtain the forecast value.

**TABLE 3:** Final CO_2_ emissions

Category	Unit	2020	2023	2024	2025	2030	2035	2040
World	Mt	32318.64	32775.54	33730.53	34713.34	38501.86	42703.85	47364.43
EU	Mt	2550.94	2520.33	2490.08	2460.20	2312.59	2284.84	2257.42
G7	Mt	7464.33	7445.40	7426.53	7407.70	7314.84	7296.29	7277.79
BRICS	Mt	14535.89	15055.69	15594.07	16151.70	18667.48	19335.02	20026.43
North America	Mt	5348.09	5328.97	5309.92	5290.94	5203.24	5184.63	5166.10
Asia	Mt	16812.48	17249.60	17698.09	18158.24	19901.43	20418.87	20949.76
OECD	Mt	10778.10	10736.95	10695.95	10655.11	10465.26	10278.79	10095.64

## 4 CONCLUSION

This paper provides statistics and analysis of data including statistics on available energy use data, total energy production and consumption, energy use and production by country (oil, gas, coal, and renewables), electricity production, and consumption, and the share of each kind of energy in electricity production. This paper provides a series of graphs and databases that visualize trends in demand and consumption. Through analysis and processing, this paper summarizes the changes in energy use and the various adjustments made to the energy mix in the development of each country region in recent decades, capturing the future trends of the country's development. In addition, through the analysis of historical data, further summaries are made from the following aspects:

1. Overall energy demand

• Energy demand and production trends in different regions
• Proportion of renewable energy in power production
• Growth rate of renewable energy
• Changes in the energy trade balance

2. Data analysis of various influencing factors

• CO_2_ emission
• Energy index performance
• Energy intensity
• Carbon dioxide intensity

3. Transformation of energy consumption structure

• Proportion of different energy sources in power production
• Primary energy use and overall energy demand
• Renewable energy development

At present, with the transformation of energy consumption structure and the promotion and use of renewable energy, it can be seen that CO_2_ emissions in various regions of the world generally show a downward trend. With the further optimization of the structure, the rate of reduction will gradually accelerate. In 2040, the dependence on fossil fuels will be reduced, but low carbon is still an important proposition. Clean energy still needs further development, and new energy sources will emerge. However, with the rapid development of energy technology and the reduction of existing energy consumption, the overall energy demand will show a downward trend, and the emergence of multiple clean energy sources will also accelerate this trend. Despite the rapid development of clean energy in Asia, due to its abundant fossil energy, it will undertake more heavy industrial development.^113^ Therefore, CO_2_ emissions in Asia have remained on the rise in recent years and may worsen in the future.^114^ However, as China proposes a series of policies to reduce CO_2_ emissions such as “carbon peak”, China's CO_2_ emissions may tend to stabilize or even decline.

By 2040, the share of renewable power generation in total power generation will increase significantly. China is expected to maintain its lead because China will have higher energy demands. Japan and China will be in the first echelon of renewable energy generation, followed by India and the United States. In 2040, the dependence on fossil fuels will be reduced, and low carbon is still an important proposition. Clean energy still needs to be further developed, and new energy sources other than existing renewable energy sources will emerge.

## FUNDING INFORMATION

This work was supported in part by the National Natural Science Foundation of China (5210071288); the National Key Research and Development Program of China (2019YFE0118000); the Guangxi Key Research and Development Program of China (2021AA11008) and the Guangxi Science and Technology Base and Talent Special Project of China (2021AC19120).

### PEER REVIEW

The peer review history for this article is available at https://publons.com/publon/10.1002/eng2.12584.

## Data Availability

Data sharing not applicable to this article. Research data are not shared.
